# Morphological abnormalities of peripheral blood cells among patients with COVID-19 disease

**DOI:** 10.1016/j.heliyon.2024.e24527

**Published:** 2024-01-17

**Authors:** Anna Karapetyan, Lyudmila Niazyan, Ruzanna Shushanyan, Tamara Abgaryan, Sevan Iritsyan, Tehmine Galechyan, Knarik Sargsyan, Anna Grigoryan

**Affiliations:** a National Centre of Infectious Diseases, Ministry of Health, Armenia; b Department of Human and Animal Physiology, Yerevan State University, 1 Alex Manoogian, RA, Yerevan, 0025, Armenia

**Keywords:** Coronavirus, Hematological abnormalities, SARS-CoV-2, Hematopoesis, Lymphocytes

## Abstract

**Purpose:**

The hematological changes in COVID-19 patients continue to receive great attention, especially in the field of public health. To our knowledge, coronavirus disease may be identified based on the severity of illness, and the study of peripheral blood smears may offer important information to facilitate the identification. Thus, we evaluated the morphological abnormalities (atypical and immature lymphocytes, lymphocytes with micronuclei, various nuclear abnormalities among erythrocytes) and quantitative changes in peripheral blood cells among 48 individuals with COVID-19 disease.

**Methods:**

The present study is a retrospective analysis of 48 individuals, including 24 hospitalized patients diagnosed with COVID-19 disease. The blood smears of all patients were subjected to a hematological examination to identify various morphological abnormalities in white and red blood cells. In addition, a micronucleus test was conducted to assess the incidence of chromosomal damage in lymphocytes. Furthermore, the complete blood count (CBC) was performed to evaluate changes in peripheral blood cells, particularly the differential total leukocyte count, which could indicate the immune response against viral infection in COVID-19 patients.

**Results:**

The findings of the hematological study conducted on patients diagnosed with COVID-19 disease revealed neutrophilia, eosinophilia, mild monocytosis, decreased hematocrit level, elevated erythrocyte sedimentation rate (ESR), and immature leukocytes. It was observed that patients who were infected with coronavirus demonstrated mild thrombocytopenia. Furthermore, the micronucleus test indicated the presence of immature cells with micronuclei among lymphocytes and numerous nuclear abnormalities in red blood cells. These results help to shed light on the hematological changes that occur in COVID-19 patients, and could potentially contribute to the development of more effective treatments for the disease.

**Conclusions:**

The examination of complete blood counts (CBCs) in conjunction with peripheral blood smears offers a potential means of identifying the impact of SARS-CoV-2 infection on the hematopoietic and immune systems, thereby providing early indications of inflammation. Based on a study, it has been suggested that SARS-CoV-2 may affect red and white blood cells causing morphological alterations thereby establishing a corresponding relationship with disease severity.

## 1 Introduction

**Fig. 1:**
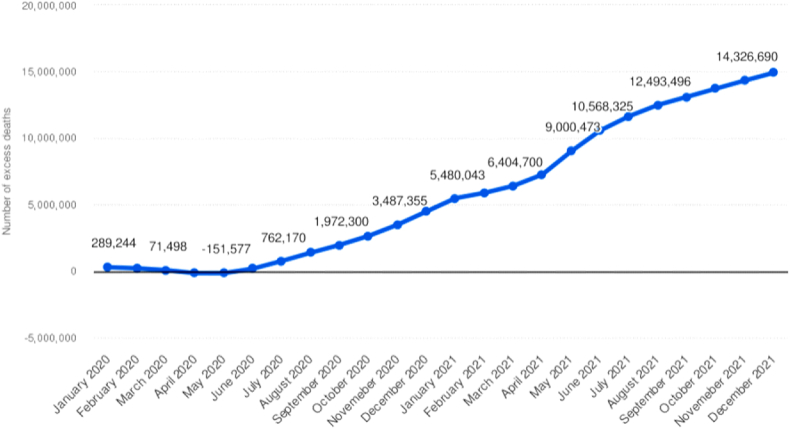
Cumulative mean number of excess deaths associated with the COVID-19 pandemic worldwide in 2020 and 2021, by month.

The coronavirus disease pandemic caused by severe acute respiratory syndrome coronavirus-2 (SARS-CoV-2) has led to global healthcare issues worldwide [1,2]. According to data from the World Health Organization, the number of individuals with this disease reached 2.1 billion in 2021, and there were about 4.5 million deaths likely due to continuous mutations of the virus and its rapid spread [3,4]. The grim statistics of COVID-19-associated deaths during 2020–2021 by month are depicted in Fig. 1. Despite the introduction of multiple vaccines and pharmaceutical treatments that effectively reduce disease severity, hospitalizations, and deaths associated with COVID-19, the pandemic continued to persist. Furthermore, it’s important to acknowledge that many individuals still suffer from short- or long-term COVID-19-associated complications, particularly those who lack access to vaccines or treatments, or who have underlying conditions that make them more susceptible to them [5,6].

Due to substantial lung tissue destruction and dysregulated immune activation, the SARS-CoV-2 infection has been linked to severe acute respiratory syndrome [[7], [8], [9], [10]]. Although it was primarily reported as a respiratory tract infection, recent studies show that COVID-19 can cause a wide range of clinical symptoms, including mild to moderate upper respiratory tract infections and severe systemic diseases that affect the immune, gastrointestinal, cardiovascular, neurological, immunological, and hematopoietic systems [11,12].

COVID-19 patients may experience a variety of blood clotting abnormalities. Several papers have described the hematological effects of SARS-CoV-2 infection, with an emphasis on leucocytes and platelets, since the start of the COVID-19 pandemic [[12], [13], [14]]. Moreover, COVID-19 triggers inflammatory processes that result in the release of immature blood cells from bone marrow, affecting the myelopoiesis system, similar to other viruses that impact hematopoiesis and the immune system in stages [15]. Likewise, individuals with various viral infections, including Epstein-Barr virus infection, CMV infection, rubella, Hantavirus infection, viral hepatitis, and HIV infection, exhibit large lymphocytes with varying shapes in their peripheral blood [16]. Researchers have identified atypical lymphocytes in peripheral blood and bronchoalveolar lavage (BAL) samples also from COVID-19 patients, which could offer valuable insights into the pathophysiology of the disease and facilitate its diagnosis or prognosis [[17], [18], [19]].

Generally, individuals with COVID-19 show abnormal results in standard peripheral blood tests [20]. Severe cases of COVID-19 usually involve a “cytokine storm” of proinflammatory cytokines, characterized by viral pneumonia patterns [21]. Moreover, recent research suggests that co-infection of bacteria and fungus may contribute to severe cases of COVID-19 [9].

Our study approached COVID-19-related complications from a hematological perspective, revealing important insights that could inform strategies for limiting the virus’s spread. The first registered cases of COVID-19 were reported in the Republic of Armenia on March 1, 2020, and the 100th case was on March 18 of the same year.[22]

In the frames of this cohort study, we conducted the prospective examination of 48 patients from which 24 individuals were diagnosed with COVID-19 disease between August and November 2020 at the Nork National Center of Infectious Disease Hospital. Our goal was to study peripheral blood cell morphology and the alongside abnormalities among COVID-19-infected patients considering that the studies covering COVID-19-associated hematological complications are scarce and barely dedicated to the individual parameters of the blood. For this purpose, the blood smears have been analyzed to identify hematological abnormalities touching upon the changes observed in lymphocytes and erythrocytes.

## 2 Materials and methods

### 2.1 Study population

This study aimed to evaluate the quantitative and qualitative changes of both RBCs and WBCs in COVID-19 patients upon their hospital admission as well as the alterations observed in levels of hemoglobin, ESR, and hematocrit.

The study involved 48 patients aged 45 to 69. They were divided into two groups: a healthy control group (n = 24) and a group of patients infected by COVID-19 (n = 24). The control group was selected based on clinical, laboratory, and physical examination data to eliminate the COVID-19 disease and any risk for cardiac, pulmonary, or related diseases, and comorbidities. Patients suspected of those diseases or associated risks were excluded from the study.

### 2.2 Management of the patient’s treatment

Since the admission of patients to the hospital, patients were provided with symptomatic treatment during the initial days of their stay, according to the WHO guidelines.[23] This included medication designed to alleviate fever (acetaminophen, ibuprofen), as well as analgesics, antiviral (remdesivir), and antimicrobial drugs (azithromycin) to prevent co-infection. In the case of any complications, such as pneumonia, antibiotics and steroids were administered, taking into account the patient’s clinical status. Patients suspected to have high coagulation levels were provided with anticoagulant medications.

### 2.3 Data collection and blood sample examination

Blood samples were promptly collected from all patients upon admission to the hospital following a positive result from the PCR test for coronavirus disease. Within the studied group, the disease was accompanied by a typical acute phase with evident symptoms.

The blood smears were obtained from all patients and collected in special vacutainers containing EDTA (ethylenediaminetetraacetic acid). These samples were processed for the complete blood count (CBS) test along with the differential total leukocyte count to evaluate the absolute count of immature leukocytes, neutrophils, eosinophils, basophils, and monocytes. In order to receive accurate results two independent hematologists were examined all blood smears to identify and describe possible morphological abnormalities observed among lymphocytes and erythrocytes. Thus, the relative number of lymphocytes with micronuclei and erythrocytes with nuclear abnormalities, such as karyorrhexis, karyolysis, nucleus protrusions, and various cellular anomalies, was determined.

The peripheral blood of healthy people, whose physiological parameters correspond to the normal range, served as a control. The collected peripheral blood streams were fixed in 96% ethyl alcohol and stained with Gimza Mein-Grunwald solutions, according to Pappenheim [24], and Shiffe reactive according to Feulgen [16]. The hematological samples were examined using a trinocular microscope (B-293, OptikamB5 Digital Camera, Italy), and Optika Liteview software was used to record the images at a magnification of×400 and ×1000.

### 2.4 Statistical analysis

All experimental data are presented as medians with interquartile ranges using Statistica 11 software for CBC results. The number of micronuclei was presented as mean ± SD. Differences between the two groups were compared using Student’s *t-*test, and *p* < 0.05 was considered statistically significant.

## 3 Results

The data presented in Table 1 reflects the complete blood count (CBC) results of 24 patients infected with COVID-19. Of these patients, 25% (6 individuals) showed nearly normal levels of hemoglobin and platelets, while the remaining 18 patients demonstrated significantly decreased levels of the aforementioned indicators along with hematocrit. The study also highlights that the number of lymphocytes, neutrophils, and eosinophils in white blood cells increased significantly in COVID-19 patients. Additionally, there was a noticeable increase in basophil numbers, although the difference was not significant between the two groups. In contrast, the number of monocytes was found to be slightly increased among individuals infected with the coronavirus in comparison to healthy controls. Furthermore, the erythrocyte sedimentation rate (ESR) were observed to be elevated. Table 1 presents an overview of the CBC results obtained from COVID-19 patients.

**Table 1:** The hematological findings of CBC test of healthy and COVID-19 patients.

Hematological results	Healthy patients (n = 24)	Patients with COVID-19 (n = 24)	NR	*p-value*
RBC (×10^6^/μL)	4.29 (3.39–5.2)	4.27 (2.71–4.81)	5.1 × 10^6^/μL	0.468
WBC (×10^3^/μL)	4.17 (1.07–7.74)*	5.27 (2.49–7.74)	4.5 × 10^3^/μL	0.14
Lymphocytes (×10^3^/μL)	0.74 (0.22–2.33)*	1.29 (0.73–2.30)	1.1–4.0	0.01
Basophiles (×10^3^/μL)	0.9 (0.61–1.52)	0.88 (0.6–1.2)	0.01	0.454
Neutrophils (×10^3^/μL)	3.77 (0.63–8.3)*	5.8 (1.68–15.91)	2.0–8.0	0.064
Eosinophils (×10^3^/μL)	0.6 (0.1–0.9)*	1.1 (0.1–2.7)	0–0.8	0.1
Monocytes (x10^3^/μL)	0.345 (0.03–0.79)*	0.445 (0.14–0.76)	0.1–0.9	0.136
Hb (G/DL)	134 (105–167)	132 (75–171)	125 g/L	0.38
ESR (mm/h)	11.58 (10–15)*	20.5 (11–40)	<12	0.005
PLT (×10^3^/μL)	236.91 (139–339)	230.16 (90–349)	275 × 10^3^/μL	0.42
Hct (%)	45.2 (30–49)*	41.5 (18–49)	25%	0.22
Immature leukocytes (%)	1.07 (0.66–1.82)*	2.11 (0.9–4.4)	0.0–1.0	0.04

Abbreviations: *RBS* – red blood cells; *WBS* – white blood cells; *Hb* - hemoglobin; *ESR* - erythrocyte sedimentation rate; *PLT* - platelet; *Hct* – hematocrit.

Data are presented as medians (interquartile range), *-p < 0.05 considered as significant.

Our research has revealed that individuals who have contracted coronavirus exhibit a decreased hematocrit level, as well as heightened erythrocyte sedimentation rate (ESR) in their blood samples, as compared to the standard range. This phenomenon may be due to the presence of anemia, which often coincides with an increase in plasma mass [25]. Our findings highlight the importance of ESR for COVID-19 patients, as the changes in ESR levels are directly linked to the shape and size of red blood cells caused by the virus infection [26], which was also examined within the study.

While ESR levels tend to rise among COVID-19 patients with pneumonia or severe disease, it cannot be relied upon as a single metric to determine the severity or prognosis of the disease [27]. It is crucial to exercise caution in interpreting ESR levels in COVID-19 patients, as it is not a definitive diagnostic indicator. Nevertheless, it can play a role in the diagnosis and prognosis of COVID-19 disease, particularly when used in conjunction with other medical indicators.

### 3.1 Morphological modifications

Studies of COVID-19 patients have reported quantitative abnormalities in their hematological variables, such as the presence of aberrantly shaped lymphocytes, as well as atypical and immature cells, which are attributed to a disturbance in myelopoiesis system functionality [15,28]. To further investigate morphological alterations in peripheral blood lymphocytes, we employed micronucleus testing. Our analysis of utilizing this testing methodology has indicated a significant increase in the number of micronuclei among lymphocytes, as presented in Table 2. Specifically, the number of lymphocytes with micronuclei was significantly high among COVID-19 patients.

**Table 2:** The results of the micronucleus test among healthy and COVID-19 patients.

Lymphocytes with micronuclei (%)	Healthy patients (n = 24)	Patients with COVID-19 (n = 24)	p-value*
	0.375 ± 0.095	1.667 ± 0.935*	<*0*.*05*

Data presented as mean ± SD, *- *p <* 0.05 shows significant differences among groups based on Student *t-*test.

It has been reported that the clinical importance of abnormal lymphocytes among COVID-19 patients hasn’t been well studied. A comparison study was conducted on COVID-19 patients, admitted to the intensive care unit (ICU) and non-ICU wards, which found that the patients' blood often had atypical lymphocytes accompanying positive PCR tests [29].

**Fig. 2:**
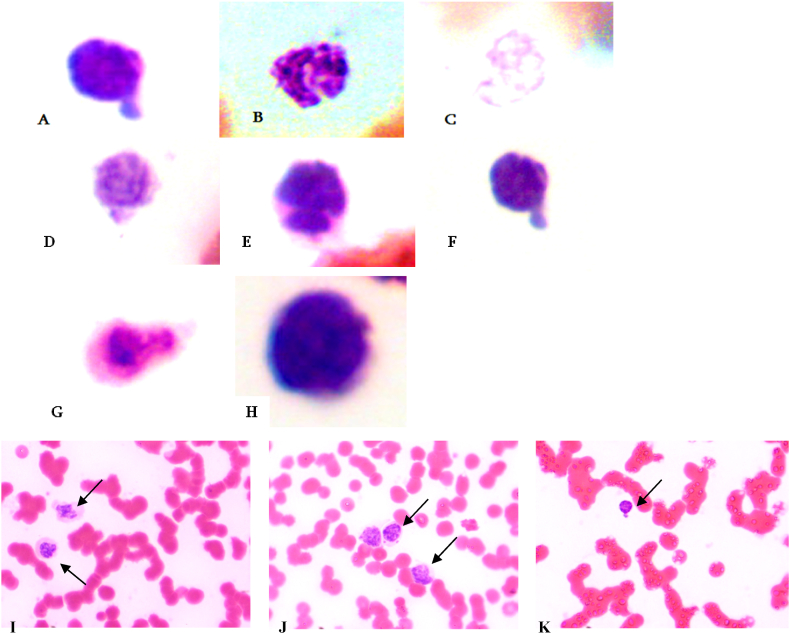
Lymphocytes with various nuclear abnormalities of the peripheral blood COVID-19 patients. A. protrusion, B. karyolysis, C. karyorrhexis, D-H, atypical lymphocytes (magnification ×1000), I. karyorrhexis, J. micronuclei within the lymphocytes, K. protrusion (magnification ×400).

Fig. 2 shows the assessment of nuclear abnormalities (A-C), and atypical lymphocytes (D-H). Notably, these morphological changes in lymphocytes in the form of protrusions, karyolysis, karyorrhexis, and prominent atypical cells were demonstrated in almost all patients (I–K).

Our analysis also revealed remarkable alterations in the size and shape of red blood cells caused by COVID-19, which is closely linked to the virus-induced anaemia. The determination of erythrocyte abnormalities under coronavirus exposure showed that the number of altered erythrocytes in the bloodstream of patients with COVID-19 disease was statistically significant for some anomalies of RBCs (Table 3).

**Table 3:** Changes in the number of different erythrocyte abnormalities in the peripheral blood smears among healthy and COVID-19 patients.

Erythrocyte abnormalities	Healthy patients (n = 24)	Patients with COVID-19 (n = 24)	Norm	p-value*
Echinocytes (%)	0.04 ± 0.03	0.10 ± 0.02*	<6	<*0*.*05*
Sickles (Hooked Erythrocytes) (%)	–	0.15 ± 0.02	*-*	<*0*.*05*
Meniscocytes (crescent-shaped erythrocytes) (%)	0.01 ± 0.01	0.17 ± 0.04*	*-*	<*0*.*05*
Schistocytes (Fragments) (%)	–	0.17 ± 0.06	*-*	<*0*.*05*
Spherocytes (%)	0.05 ± 0.03	0.09 ± 0.06*	<1	*>0*.*05*
Stomatocytes (%)	0.17 ± 0.03	0.17 ± 0.04*	<6	*>0*.*05*
Elliptocytes (%)	–	0.14 ± 0.04	*-*	<*0*.*05*

Data presented as mean ± SD, *-*p* < 0.05 shows significant differences among groups.

**Fig. 3:**
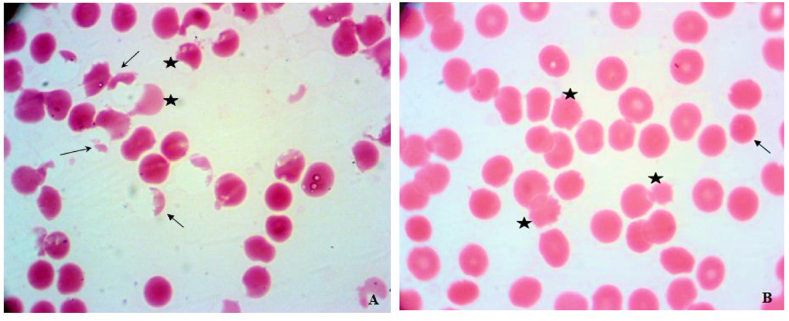
Erythrocytes with various modifications in the peripheral blood smear of COVID-19 patients. **A.** sickles (asterisk), schistocytes (fragments) (arrows), meniscocytes (crescent-shaped erythrocytes) (arrowhead), **B.** echinocytes (asterisk), spherocytes (arrowhead).

In our study, various types of morphological disorders of erythrocytes were determined in 50% of the patients, including echinocytes, sickles (meningocytes), meniscocytes (crescent-shaped erythrocytes), spherocytes, stomatocytes, ellipocytes (Fig. 3A and B). The number of observed abnormalities showed a significant increase. Albeit, for spherocytes and stomatocytes the changes in number were not significant among studied groups.

It is noteworthy that the presence of all these abnormal erythrocytes may indicate autoimmune hemolytic anemia and elevated ESR, which is due to the disruption of the normal cell structure and the functioning triggered by the virus.

The observed morphological aberrations of blood cells, such as modifications in the size and shape of erythrocytes and the appearance of atypical lymphocytes, collectively hint at an adaptive and compensatory immune response to coronavirus disease. These changes signify an attempt by the immune system to combat the pathogenic impact of the virus.

Such observations may also be attributed to the disturbance of normal granulopoiesis, potentially resulting from the cytokine storm and hyperinflammation that display morphological heterogeneity particularly in lymphocytes even after antiviral treatment [30].

## 4 Discussion

The present study has elucidated distinct morphological alterations in the peripheral blood cells of individuals afflicted with COVID-19. Prior research endeavors have documented some morphological aberrations linked to severe COVID-19 infection. Our study primarily aims to provide an analysis of the morphological alterations in the peripheral blood cells of patients diagnosed with COVID-19 in the acute phase of the disease, with a special focus on a particular morphological aberration observed in the lymphocytes and RBCs. In fact, very few blood smears prepared from COVID-19 patients have been published so far. Almost all described RBC abnormalities can be recognized in some case reports, which, however, focused on the morphology features of white cells during COVID-19 [31]. However, the current study demonstrates that lymphocytes among COVID-19 patients are characterized by an immature configuration and the presence of micronuclei. Additionally, red blood cells also exhibited diverse morphological abnormalities which are presented in the frames of this study.

The study’s findings substantiate the notion that COVID-19 patients during the acute phase of disease development experience lymphopenia, mild thrombocytopenia, higher leukocyte counts, and elevated erythrocyte sedimentation rate (ESR).

Morphologically, the study identifies numerous atypical lymphocytes, as well as the lymphocytes with micronuclei, and an increased count of abnormal red blood cells, including echinocytes, sickle cells, meniscocytes, schistocytes, spherocytes, stomatocytes, and ellipocytes. Notably, the study also highlights that the levels of hemoglobin (Hb), basophils, and platelets (PLTs) have been impacted, albeit these changes were not found to be significant compared to the healthy controls. These results contribute to the growing body of literature on the pathophysiology of COVID-19 and its effects on the immune and hematopoietic systems.

Retrospective cohort studies have demonstrated that changes in number of lymphocytes observed among patients infected with coronavirus can be an indicator of patient outcomes, with differentiation of peripheral white blood cells potentially signaling immunologic dysfunction in the early stages of the disease [32]. In some studies, the lymphocyte count is proposed as an indicator for classification of disease severity alongside pulmonary imaging as the main test for classification of disease type [33]. However, the cause and the exact mechanisms of increased white blood cell count in COVID-19 patients remains unknown [34].

The coronavirus has been found to affect not only lymphocytes, but also red blood cells at the cellular level. This occurs due to oxidative stress, which can compromise the integrity of red blood cell membranes, rendering them more susceptible to attack by reactive oxygen species (ROS). As a consequence, these cells may undergo lysis and lose their ability to carry oxygen, resulting in dysregulation of erythrocyte metabolism and decreased levels of RBCs. Furthermore, SARS-CoV-2 has been shown to impair heme metabolism and cause hemoglobin cleavage, ultimately thinning the membrane of erythrocytes [[35], [36], [37]], leading to abnormal changes in their morphology. ROS-induced damage to hemoglobin functionality is also known to cause anemia and a multi-faceted syndrome in COVID-19 [38].

While data on blood cell morphology in COVID-19 patients is limited, anomalies in red blood cell morphology have been identified, suggesting the presence of immuno-inflammatory-mediated stress erythropoiesis [39]. Hematological evaluation of peripheral blood film is therefore considered a crucial technique for determining COVID-19 severity, as morphological anomalies can corroborate diagnosis and identify infection in hospitalized patients.

Our study enhances understanding of peripheral blood symptoms in COVID-19 patients with morphological abnormalities for both WBCs and RBCs, potentially shedding light on the disease’s pathogenesis and informing future research, even with a small patient sample size. Nonetheless, further investigations are warranted to elucidate the exact mechanisms underlying these hematological changes and their role in COVID-19 pathogenesis.

## 5 Conclusion

Our findings suggest that the individuals with COVID-19 exhibit varying levels of ESR and WBCs, as well as RBCs with distinctive morphological disturbances in comparison with the healthy controls. The SARS-Cov-2 virus appears to trigger specific morphological abnormalities accompanied by an increase in sickle cells, meniscocytes, elliptocytes, schistocytes, and echinocytes among RBCs and nuclear disorders with immature cells among lymphocytes. These results suggest potential cytotoxic effects of the virus on the hematopoietic system functionality. Although the findings of the CBC analysis in COVID-19 patients were associated with deviations from normal ranges and morphological abnormalities, the modifications of blood cells in COVID-19 patients require additional and in-depth investigation. Detecting these morphological abnormalities in peripheral blood smears alongside the clinical data could be beneficial in predicting and diagnosing the severity of COVID-19 disease. Consequently, understanding the response of the hematopoietic system against the virus is critical. The results of this study will provide valuable data into the hematological manifestations of COVID-19 and the possible underlying mechanisms.

## 6 Data limitation

This study has limitations regarding the small number of patients and the adult age that can possibly compound the hematological parameters along with the demographic features. However, we believe that the hematological findings demonstrated in the manuscript can be useful and valuable for future studies about COVID-19-associated complications. Therefore, more in-depth studies are needed with a large number of patients in order to verify the study outcomes.

## Ethics statement

The whole study was performed by the Declaration of Helsinki (1975) and approved by the Ethics Committee of the Health Research and Development Initiative of Armenia (TBRPC-ERC-2020-001, 5 January 2020).

## Informed consent statement

Patient consent was waived due to the retrospective nature of the study and the NICH provided consent for the use of patient’s data upon admission.

## Data availability statement

As the data used in this study is obtained from a hospital, we are obligated by the hospital policies to safeguard patient privacy and confidentiality. However, derived data that supports the study’s findings can be made available upon request from the corresponding author.

## CRediT authorship contribution statement

**Anna Karapetyan:** Validation, Supervision, Investigation, Funding acquisition, Data curation, Conceptualization. **Lyudmila Niazyan:** Resources, Project administration, Investigation, Formal analysis, Data curation. **Ruzanna Shushanyan:** Writing – review & editing, Writing – original draft, Validation, Resources, Investigation, Data curation. **Tamara Abgaryan:** Software, Resources, Formal analysis. **Sevan Iritsyan:** Resources, Methodology, Formal analysis, Data curation. **Tehmine Galechyan:** Validation, Resources, Methodology, Formal analysis, Data curation. **Knarik Sargsyan:** Validation, Resources, Methodology, Formal analysis, Data curation. **Anna Grigoryan:** Writing – review & editing, Software, Methodology, Formal analysis, Data curation, Conceptualization.

## Declaration of competing interest

The authors declare that they have no known competing financial interests or personal relationships that could have appeared to influence the work reported in this paper.
